# Social Isolation Induces Neuroinflammation And Microglia Overactivation, While Dihydromyricetin Prevents And Improves Them

**DOI:** 10.21203/rs.3.rs-923871/v1

**Published:** 2021-10-01

**Authors:** Alzahra J Al Omran, Amy S Shao, Saki Watanabe, Zeyu Zhang, Jifeng Zhang, Chen Xue, Junji Watanabe, Daryl L. Davies, Xuesi M. Shao, Jing Liang

**Affiliations:** University of Southern California School of Pharmacy; Western Michigan University; University of Southern California School of Pharmacy; University of Southern California; University of Southern California School of Pharmacy; University of Southern California; University of Southern California School of Pharmacy; David Geffen School of Medicine at UCLA; University of Southern California School of Pharmacy

**Keywords:** Social isolation, neuroinflammation, microglia, anxiety, GABAAR, dihydromyricetin (DHM)

## Abstract

**Background:**

Anxiety disorders are the most prevalent mental illnesses in the U.S. and are estimated to consume one-third of the country’s mental health treatment cost. Although anxiolytic therapies are available, many patients still exhibit treatment-resistance, relapse, or substantial side effects. Further, due to the COVID-19 pandemic and stay-at-home order, social isolation, fear of the pandemic, and unprecedented times, the incidence of anxiety has dramatically increased. Previously, we have demonstrated dihydromyricetin (DHM), the major bioactive flavonoid extracted from *Ampelopsis grossedentata*, exhibits anxiolytic properties in a mouse model of social isolation-induced anxiety. Because GABAergic transmission modulates the immune system in addition to the inhibitory signal transmission, we investigated the effects of short-term social isolation on the neuroimmune system.

**Methods:**

Eight-week-old male C57BL/6 mice were housed under absolute social isolation for 4 weeks. The anxiety like behaviors after DHM treatment were examined using elevated plus maze and open field behavioral tests. Gephyrin protein expression, microglial profile changes, NF-κB pathway activation, cytokine level, and serum corticosterone were measured.

**Results:**

Socially isolated mice showed increased anxiety levels, reduced exploratory behaviors, and reduced gephyrin levels. Also, a dynamic alteration in hippocampal microglia were detected illustrated as a decline in microglia number and overactivation as determined by significant morphological changes including decreases in lacunarity, perimeter, and cell size and increase in cell density. Moreover, social isolation also induced an increase in serum corticosterone level and activation in NF-κB pathway. Notably, DHM treatment counteracted these changes.

**Conclusion:**

The results suggest that social isolation contributes to neuroinflammation, while DHM has the ability to restore neuroinflammatory changes induced by anxiety.

## Background

Anxiety disorders are the largest class of mental health diseases in the U.S. and are estimated to consume one-third of the country’s mental health spending. Considering the ineffective therapeutics in the clinic due to treatment resistance, inconsistent patient adherence, and other exogenous factors, the prevalence of chronic anxiety continues to be on the rise (1–3). The rise in individuals suffering anxiety has been particularly noticeable during the ongoing COVID-19 pandemic that has resulted in quarantine and social isolation (4). The pandemic also has led to an increase in posttraumatic stress disorder (PTSD) (5). The pandemic is bringing up similar fears and mental distress, due to loss of family member(s) and loved ones (5, 6). The ongoing increase in cases of individuals suffering from anxiety disorders, coupled with the lack of effective medications for many, highlight the need for new treatment strategies to prevent or manage anxiety disorders.

Clinical evidence indicates that GABAergic neurotransmission alteration involves in pathophysiology of anxiety disorders in humans (7). Therefore, modifying GABA_A_ receptor (GABA_A_Rs) activity is a target for regulating anxiety (7, 8). We have demonstrated that dihydromyricetin (DHM) [(2R,3R)-3,5,7-trihydroxy-2-(3,4,5-trihydroxyphenyl)-2,3-dihydrochromen-4-one], a major bioactive flavonoid extracted from *Ampelopsis grossedentata*, is a positive allosteric modulator (PAM) of GABAergic transmission, and thus has the potential to regulate anxiety-like behavior via its action on GABAergic receptors (9, 10). In addition, we found that DHM antagonizes the acute and chronic effects of alcohol on GABA_A_Rs (9, 10). Thus, the activity of DHM on GABA_A_Rs provides one possible mechanism for its role in anxiolysis (10). Because GABAergic transmission modulates the immune system in addition to the inhibitory signal transmission, we investigate the effects of social isolation on the neuroimmune system. To date, the role of neuroinflammation in anxiety pathogenesis is not well established. Several studies had highlighted the regulatory role of GABA in neuroimmune functions. GABA involves in inflammation regulation by modulating the production of pro-inflammatory cytokines through activating inflammatory signaling pathways such as nuclear factor-κB (NFκB) and mitogen-activated protein kinase (MAPK) (11, 12). Indeed, GABAergic system components, including GABA enzyme, transports, and receptors, are expressed in the immune cells (13, 14).

The extended psychological stress that occurs due to social isolation disrupts the hypothalamic-pituitary-adrenal (HPA) axis, which is considered the primary stress adaption pathway in the body (15). This disruption has the potential to increase proinflammatory cytokines, persistent microglia and astrocytes activation, and reduce synaptic plasticity (16). Additionally, several studies indicated that GABAergic deficits during stress contribute to HPA hyperactivity that correlated with anxiety pathogenesis (17, 18). The outcome of these immunoendocrine dysregulations could lead to the occurrence of neuroinflammation and anxiety.

Microglia, the resident macrophages of the brain’s innate system, are a key player in modulating the neuroinflammatory response in the CNS. Microglia are not only involved in the nervous system infection and debris phagocytosis, but also play a crucial role in the physiological development of the brain by engaging in the shaping process of neuronal circuits and synapse plasticity (19, 20). During various psychological stressors such as social isolation, microglia undergo several changes that compromise their functions and further lead to neurological and neurodegenerative disorders (21). To understand the role of microglial in social isolation-induced anxiety, we utilized a social isolation mouse model that induces anxiety via reduction of social interaction as a stressor. The primary goal of this study was to determine whether social isolation-induced anxiety leads to neuroinflammation, and to understand the pharmacological mechanisms of DHM as an alternative therapy for preventing and reducing neuroinflammation.

## Methods

### Animals and treatments

#### Overview:

Eight-week-old male C57BL/6 mice (Charles River Laboratories, Hollister, CA) were housed in the vivarium under a 12 h light/dark cycle with direct bedding and free access to food and water. All animal experiments were performed according to the protocols approved by the University of California, Los Angeles (UCLA) and the University of Southern California (USC) Institutional Animal Care and Use Committees, and all methods were carried out in accordance with relevant guidelines and regulations. Animals were habituated to the vivarium for 2 days before beginning experimentation.

#### Social isolation:

Social isolation is reported to elicit anxious and depressive behaviors in rodents (22–25). For the present study, we modified these methods to induce stress associated with social isolation by using opaque-walled cages, thus depriving the animals of environmental enhancers (e.g., toys, objects, etc.). We investigated anxiety-like behaviors after 4 and 6 weeks post-social isolation to determine behavioral responses comparable to the established 4–6 weeks isolation that results in anxiety (24). We used these time points to determine the potential therapeutic effects of DHM. Group-housed mice were housed with the standard 3–4 mice per cage. Isolated mice were singly housed with opaque walls without human handling except to change cages once per week.

The mice were divided as follows:

1. Group-housed mice without any drug administration for 2 weeks, and then given daily sucrose agar as a vehicle for an additional 2 weeks (G2+Veh2).
2. Group-housed mice without any drug administration for 2 weeks, and then given daily DHM in sucrose+agar for an additional 2 weeks (G2+D2, 2mg/kg DHM).
3. Isolated mice without any drug administration for 2 weeks, and then given daily sucrose agar as a vehicle for an additional 2 weeks for a total isolation period of 4 weeks (Iso2+Veh2).
4. Isolated mice without any drug administration for 2 weeks, and then given daily DHM for an additional 2 weeks for a total isolation period of 4 weeks (Iso2+D2).
5. Isolated mice without any drug administration for 4 weeks, and then given daily vehicle for an additional 2 weeks for a total isolation period of 6 weeks (Iso4+Veh2).
6. Isolated mice without any drug administration for 4 weeks, and then given daily oral administration of DHM for an additional 2 weeks for a total isolation period of 6 weeks (Iso4+D2).

#### Drug preparations:

DHM (HPLC purified ≥ 98%, Master Herbs Inc., Pomona, CA) was given orally as agar cube once per day (2 mg/kg) for 2-weeks (10). To prepare the DHM or vehicle agar cube, 3% agar was prepared with water, heated to ~90 °C to dissolve the agar, then DHM + 5% sucrose or 5% sucrose only were added and mixed until cooled and solidified. Agar was prepared for the mice by cutting it into cubes of 0.5 × 0.5 × 0.5 cm each.

#### Drug administration:

Every evening (2 PM), all food from the cages of each mouse was removed, and an agar cube was placed in the cage for each mouse. The mouse was observed to ensure it ate the agar cube, which ranged from 30 to 90 minutes. Afterward, 4g of regular rodent food (the recommended daily amount for an adult mouse; was placed in the cage for each mouse for the rest of the day (26). To ensure each mouse of the group housing mice received one cube, they were isolated, fed, and then returned to group housing.

#### Behavioral Testing:

Anxiety-like behavior was tested 24 h after the last treatment, at the end of the 4-week or 6-week time points in the following evening (8 PM; in the dark phase of 12/12-hr light/dark cycle). Anxiety tests were reliant on ethologically appropriate behavior and sensitive to ‘state’ anxiety measurements. Because mice are nocturnal animals, behavior tests were conducted during their active time to ensure accurate behavioral responses and minimize interference of their circadian cycle. Behavioral tests were performed under indirect red lighting and recorded with a video camera. Indirect red lighting was used to better assess parameters of anxiety without influencing mouse behavior (i.e., reduced activity in the open field test) and stress (27). Investigators were blind to experimental groups when conducting behavioral analyses.

#### 1. Elevated plus maze:

The elevated plus maze was conducted following a previously published protocol (28). The elevated plus-maze apparatus was made of opaque, 0.6 cm-thick plastic. It comprised of two open arms 25×8 cm across from each other and perpendicular to two closed arms 25×8 cm with a center platform of 8×8 cm. The closed arms had a 20 cm-high wall that enclosed the arms. The walls and floors of the closed arms were black, while the open arms were white. The apparatus was elevated 50 cm above the floor. Throughout the test, each animal was placed in the center of the maze facing an open arm and allowed to explore for 5 mins. The behaviors were recorded by a ceiling-mounted camera. Entry into an arm of the maze was defined by the placement of at least 3 paws into that compartment. The following measures were physiological scoring system: number of entries into open arms, closed arms, or center platform and time spent in each of these areas. All scoring was performed offline in a double-blind manner.

#### 2. Open field:

The open field test was conducted following a previously published protocol (10, 29). The open field chamber measured 50 cm (length) × 50 cm (width) × 38 cm (height) and was made from a white acrylic plastic sheet. 4 × 4 grid lines were drawn to divide the floor into 10 × 10 cm squares, and an additional 20 × 20 cm square zone was drawn in the center. Mouse activity was assessed as previously reported in Chen et al., 2004 for 10 min. The following parameters were summed for each animal during the 10-min test: the time spent in the central zone, time spent in the 4-corner square grid, pathlength (cm) traveled in the apparatus (determined by measuring the distance of the nose of the mouse relative to the 10 × 10 cm square grid lines on the floor of the open field chamber), and the numbers of times the animal reared. All scoring was conducted manually in a double-blind manner, with each recording being observed three times to minimize error.

#### Immunohistochemistry analysis:

Mouse brain was fixed in 4% formaldehyde for 24 hours, and then incubated in 30% sucrose until tissues are sink. Fixed brain was flash frozen using pre-cooled isopentane (−78 °C), sectioned at 30 μm using Microm HM525 Cryostat (Thermo) and picked up on Superfrost Plus slides (VWR, 48311–703). Sections were blocked with 5% normal goat serum and washed in PBS with 1% bovine serum albumin (BSA) and incubated with rabbit- anti-mouse Iba-1 primary antibody (FUJIFILM Wako Pure Chemical Corporation 019–19741, 1:500) or rabbit- anti-mouse Iba-1 primary antibody Alexa Fluor^®^ 594 conjugate (Cell Signaling, 48934, 1:50) overnight. Sections were washed with phosphate buffer with 1% Tween 20 (PBS-T), and then incubated in goat anti-rabbit IgG (H+L) secondary antibody (Vector laboratory CY-1300, 1:250) at room temperature for 1 hour when unconjugated antibody was used. Afterword, sections were washed three times with PBS-T followed by mounting on coverslip using Vectashield DAPI (4′6-diamidino-2-phenylindole 2HCl, Vector Labs, Burlingame, U.S.) mounting media to detect nuclei.

#### Imaging and Analysis:

The immune-stained sections were scanned for high-resolution images by Cytation 5 cell imaging multi-mode reader (BioTek, Winooski, VT, USA) or super-resolution images by Zeiss LSM880 with Airyscan confocal laser scanning microscope (Carl Zeiss Microscopy, White Plains, NY). The images from the CA1 and CA2 of the brain hippocampus were processed by Zen black and blue imaging analysis software (Carl Zeiss Microscopy) and analyzed by FracLac box counting and convex hull analysis to evaluate the morphological changes of microglia cells using ImageJ software by following the steps published in Young & Morrison, 2018 (30). Microglia cells count, within the region of interest ROI (0.3 × 0.4 mm) in the CA1 and CA2 hippocampus area, was done manually blinded to the treatment group using ImageJ cell count command and presented in cells per 1 mm^2^.

#### Western blot analysis:

Hippocampus was homogenized in pre-cooled Tris-EDTA with 1 % PMSF (0.1 M Tris-acetate buffer + 2 mM EDTA, pH 7.75). The homogenate was centrifuged at 10,000 × g for 15 minutes at 4°C and the supernatant was collected. The supernatant protein was quantified using the BCA Protein Assay kit (Pierce Biotechnology, Rockford, IL) according to the manufacturer’s instructions. 50 μg of protein was separated on a 10 % sodium dodecyl sulfate-polyacrylamide gel electrophoresis and then transferred to PVDF membranes (Bio-Rad Laboratories, Hercules, CA). The transferred membrane was blocked with a blocking buffer containing 5% skim milk in 1X Tris-buffered saline with Tween 20 (TBST) for 1 hour at room temperature. The membrane was incubated in the following primary antibody for either: rabbit anti-mouse Gephyrin (Cell Signaling 14304, 1:1000), mouse anti-mouse β-actin (Cell Signaling 4970, 1:1000), rabbit anti-mouse NF-κB p65 (Cell Signaling 8242, 1:1000) or rabbit anti-mouse Phospho-NF-κB p65 (Cell Signaling 3033, 1:1000) in 1X TBST overnight at 4°C with gentle agitation. The membrane was washed three times with 1X TBST for 10 min each and incubated with a secondary antibody goat anti-rabbit IgG or goat anti-mouse (Bio-Rad 1706515 and 1706516) in 1X TBST for 1 hour. Finally, the membrane was visualized with enhanced chemiluminescence detection reagent (Bio-Rad 1705061) and Chemi-Doc (Bio-Rad) imaging device.

#### Cytokines profile arrays:

Serum samples were pooled from 4–5 mice/group (n=4 vehicle grouped housed, n=4 DHM grouped housed, n=5 vehicle social isolated, n=5 DHM in social isolated group). According to the manufacturer’s instructions, pooled sera were tested by Profiler Mouse Cytokine Array Kit, Panel A (ARY006, R&D Systems, Minneapolis, MN, USA) for a quantitative measure of the peripheral cytokines and chemokines level to determine changes in response to the social isolation-induced anxiety. The membrane immunoreactivity was detected after adding chemiluminescence reagent mix. Array images were obtained using (Chemi-Doc imager system (Bio-Rad). The results were analyzed using ImageJ software and expressed as a mean change in the gray value relative to the vehicle grouped housed groups.

#### ELISA analysis:

Fresh blood was collected from the right atrium quickly after the animals were sacrificed. serum samples were collected by centrifugation at 1000 × *g* for 10 min at 4°C. A competitive ELISA kit (Abcam, ab108821) was used to determine the corticosterone levels according to the manufacturer’s protocol. The intensity of the color developed was measured using Synergy H1 Hybrid Multi-Mode Reader (BioTek).

### Statistical analysis

All assays were performed at least three times. All behavioral records were observed and analyzed in a double-blind manner. The data were presented as the mean ± standard error of the mean using GraphPad Prism 9 (GraphPad Software, Inc., La Jolla, CA). One or two-way analysis of variance (ANOVA) followed by Holm-Sidak multiple comparison tests were performed, the significance level is set at p < 0.05.

## Results

### DHM treatment ameliorates social isolation induced anxiety-like behaviors

**Figure 1:**
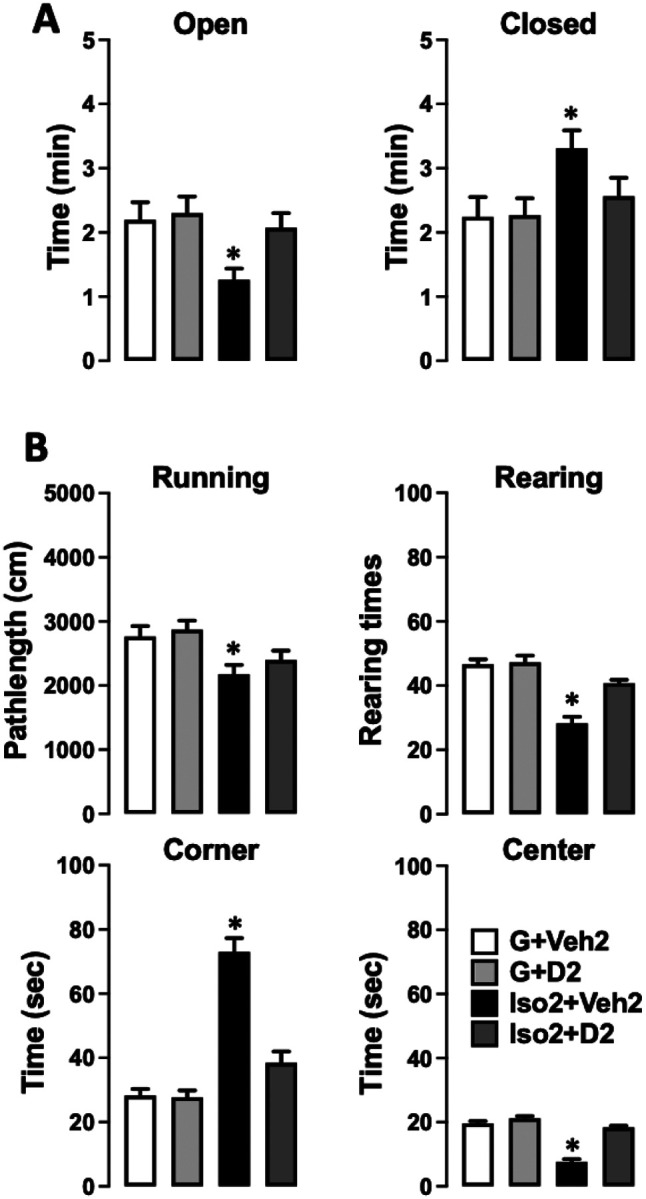
DHM reduces social isolation-induced anxiety. A. Effects of social isolation and treatment with DHM on anxiety-like behaviors as measured by the time (min) spent in the open and closed arms of the elevated plus maze. B. Effects of social isolation and treatment with DHM on locomotor activity, exploratory behaviors as measured by running distance (total distance of moving), rearing (total number of times of rearings), corner (the total duration the mouse stayed in the 4 corner 10×10 cm squares), and center time (the total time duration the mouse stayed in the center 20×20 cm square), in the open field assay. One-way ANOVA followed by multiple comparison, Holm-Sidak method to the control. For running length, P < 0.001; For numbers of rearings, P < 0.001. For stay in corners P < 0.001. For stay in the center, P < 0.001. * = p≤0.05 vs. vehicle group housing control (G2+Veh2). (n = 10–11/group).

Using a mouse model of social isolation stress, we examined social isolation stress induced anxiety levels and the effects of DHM with the elevated plus-maze (EPM) and open field (OF) tests (31–33). Group-housed control mice (G2+Veh2) spent 2.31±0.27 (min) of a total 5 min in open arms of the elevated plus-maze (Fig 1A) and 2.24±0.31 min in the closed arm. Mice socially isolated for 2 weeks, followed by 2 weeks of vehicle treatment (Iso2+Veh2) spent substantially less time (1.26±0.17 min) in the open arms compared to the closed arms (3.31±0.27 min). Socially isolated mice treated with DHM (Iso2+D2) resulted in greater time spent in the open arm (2.07±0.22 min) when compared with untreated socially isolated mice (Table S1). These results suggest that social isolation increased anxiety levels. Furthermore, DHM administration ameliorates isolation-induced anxiety behaviors, as observed with increased entry into and staying in the open arms.

To further examine anxiety-like behavior, locomotor activity, and exploration behavior were analyzed by measuring the distance traveled of the mice in the open field test (Fig 1B). During the 10 min observation trial, group-housed mice (G2+Veh2) traveled 2765±161 cm, while isolated mice (Iso2+Veh2) traveled a significantly reduced (2176±145 cm) pathlength suggesting that social isolation decreased motor activity of these mice. In contrast, isolated mice treated with DHM (Iso2+D2) resulted in greater running distance (2398±147 cm). The number of rearing and time spent in the center of the open field was significantly decreased in mice housed in isolation compared to that of group-housed mice (46.6±1.5 times vs 28.3±2.1 times) (19.6±0.7 vs 7.6±0.86). Social isolation mice spent more time in the corners compared to group-housed mice (73±4.2 sec vs 28.2±2.06 sec). Administration of DHM in Iso2+D2 increased the number of rearing (40.7±1.03 times) and the time in the center (18.3±0.49 sec) while decreasing stay in the corner duration (38.5±3.5 sec) (Table 1S). Collectively, these results suggest that isolation decreased exploratory/locomotor activity in adult male C57BL/6J mice and that DHM treatment ameliorates these behavioral responses in socially isolated mice.

### Social isolation reduces gephyrin protein expression, while DHM treatment ameliorates it

**Figure 2:**
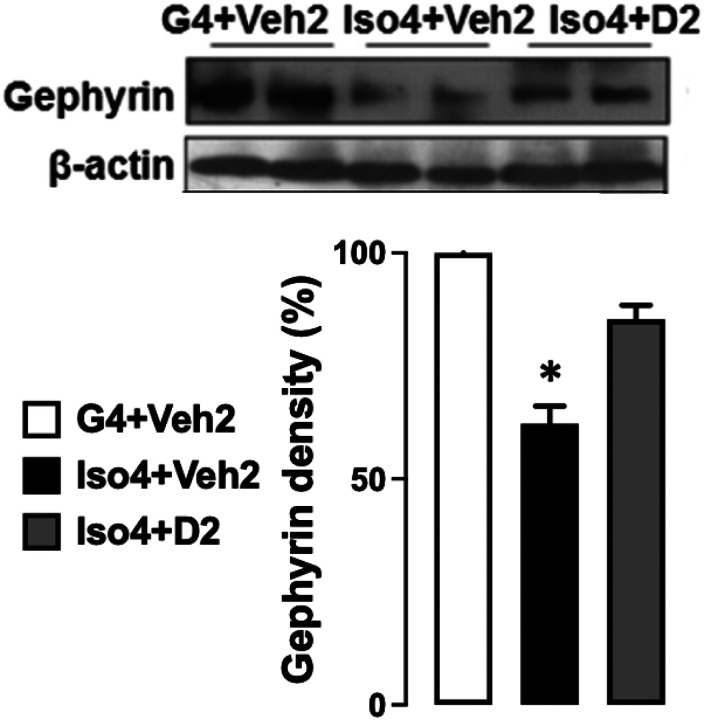
Changes in gephyrin protein expression after social isolation and the effect of DHM treatment. Grouped-house (G4+Veh2), single-housed mice (Iso4+Veh2), and single-housed with DHM treatment (Iso4+D2). β-actin from the same blot was used as a loading control. One-way ANOVA followed by multiple comparisons, Holm-Sidak’s method; * = p≤ 0.05 vs. group housing control (G4+Veh2), n=3/group. Group-housed animals are set as 100.0.

Gephyrin is a scaffold protein essential for GABA_A_R clustering via several mechanisms (34, 35). The changes in the expression of gephyrin can partially explain the changes in GABAergic neurotransmission. Gephyrin protein expression was measured in extracted hippocampi and evaluated by Western blot. Gephyrin expression was 40% lower in isolated mice compared to group-housed mice (Fig. 2) (Table 2S). DHM treatment restored gephyrin expression levels at Iso4+D2 group relative to isolated mice without DHM. Collectively, the behavioral assay and the gephyrin results confirm the phenotype of the social isolation animal model. The results also suggest that DHM pharmacologically restores gephyrin levels in hippocampi and subsequent GABA_A_R function.

### Social isolation induces loss and dystrophy in the hippocampus microglia, while DHM reverses it

**Figure 3:**
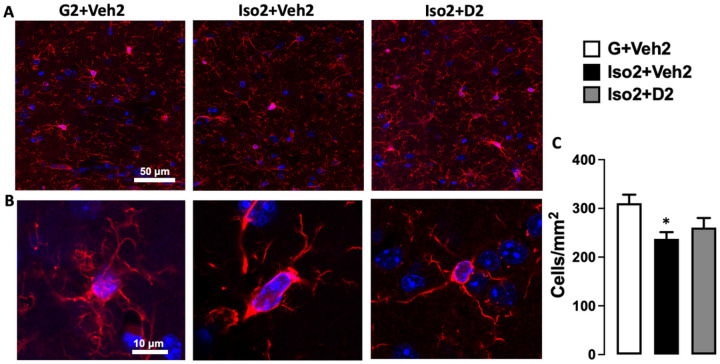
The effects of social isolation-induced anxiety and DHM treatment on microglia activation and proliferation in the hippocampal CA area. A. Representative images showing the effect of social isolation-induced anxiety on the number of labeled microglia in the hippocampus, Iba-1 (red), DAPI (blue). B. Confocal single-cell microglia images for the G2+Veh2, Iso+Veh2, and Iso+D2 obtained using a 63X oil-immersion objective. C. Quantification analysis of the microglia number in CA1 and CA2 area, data presented as the number of cells per 1mm2. One-way ANOVA followed by Sidak multiple comparisons test was used for statistical analysis, values represented as mean± SEM, * = p ≤ 0.05, n=5.

**Figure 4:**
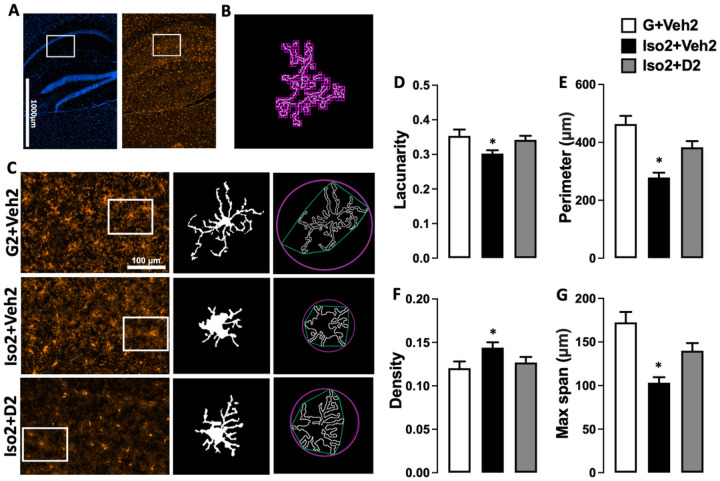
DHM treatment modulates microglia morphology in the CA1 and CA2 area of hippocampus. A. Representative photos of the hippocampus CA1 where the white rectangular are regions for photomicrograph analysis, stained for DAPI (blue, left panel) and Iba-1 (red, right panel) from the control group. B. Illustration for the box-counting method used for lacunarity calculation. C. representative images for binary microglial and convex hull (green) and enclosing circle (pink) that were used to calculate density, perimeter, and maximum span across the hull, and the corresponding microglial in each group. D. Microglial morphology profile illustrated in lacunarity, cell perimeter and density (E and F), and the maximum span across the convex hull (G). One-way ANOVA followed by Sidak multiple comparisons test was used for statistical analysis, values represented as mean± SEM, * = p ≤ 0.05.

Microglia number was reduced after short term social isolation in the CA hippocampus brain region in addition to alteration in morphological characteristics of microglia cells. The changes appeared due to the activation of cells toward more dystrophic morphology and apoptosis leading to decrease in microglia number compared to the basal state. Microglia counts in social isolation group (Iso2+Veh2) was lower (28.50± 1.64) compared to the control group G2+Veh2 (37.27 ± 2.1) (Fig. 3C). Moreover, microglia cells in Iso2+Veh2 show a significant decrease in the lacunarity value from 0.353 ± 0.018 to 0.302 ± 0.009 (Fig. 4B) as an evidence of microglia activation that was restored after DHM treatment. Lacunarity measures cell shape heterogenicity and changes in the soma (36). Moreover, a decrease in microglial cell area was observed in the social isolation group (Iso2+Veh2) illustrated in the reduction of the perimeter of the individual cell outline, where social isolation for 2 weeks followed by 2 weeks of DHM treatment (Iso2+D2) showed an increase in cell perimeter from 278.7±16.76 to 382.9±21.56 μm [F (2, 28) = 17.72, p=<0.0001] (Fig. 4C). Iso2+Veh2 microglia cells demonstrated a more compact shape illustrated in significantly higher cell density [F (2, 27) = 3.388, p=0.0387) (Fig. 3D]. In contrast, the DHM isolated group (Iso2+D2) showed higher cell density which was statistically similar to the control grouped house (G2+Veh2). In addition, we evaluated the maximum distance between two points across the convex hull and found that DHM administered social isolated group (Iso2+D2) had an average distance relatively similar to the control group house (G2+Veh2) (Fig. 4E), while the socially isolated group (Iso2+Veh2) had a substantially smaller average distance across the convex hull from 172.5±12.04 to 103.2±6.40 μm (F (2, 28) = 14.36, p=<0.0001) (Table 3S). These results suggest a modulation in several microglia morphological parameters related to the microglia activation after social isolation, indicating an acute response to social isolation challenges, which were ameliorated by DHM administration.

### Social isolation anxiety increases serum corticosterone level, activates NF-κB signaling pathway, and increases proinflammatory cytokines, while DHM attenuates these changes.

**Figure 5:**
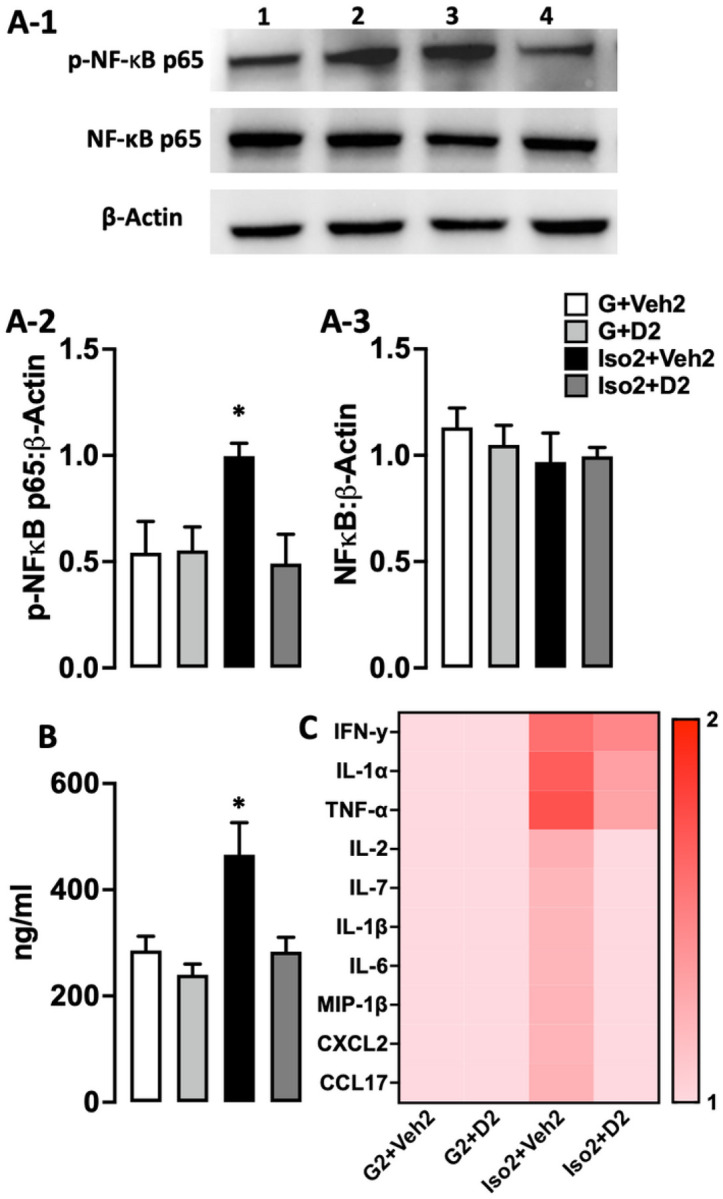
The effect of DHM on the level of serum corticosterone, p-NF-kB p65 protein expression, and proinflammatory cytokines expression after social isolation. A1. Representative Western blots of Phospho-NF-kB p65 (65 kDa), NF-kB p65 (65 kDa), and β -actin (42 kDa). (A2 and A3) Quantitative analysis ratio for the protein expression of Phospho-NF-κB p65 and NF-κB p65 with the loading control β-actin F (4,10) = 1.485 p=0.5885. B. Serum level of corticosterone (ng/ml) after social isolation (Iso2+Veh2) and following DHM treatment 2 mg/kg and 10 mg/kg. One-way ANOVA followed by multiple comparisons, Holm-Sidak’s method. C. Heat map of serum cytokines and chemokines level. The color key indicates response level ranging from the highest (red) to the lowest (pink). Data represented as the inverted mean gray value expressed in each cytokine/chemokine. Each data point was normalized to the baseline value (G2+Veh2). (G2+Veh) grouped housed vehicle, (G2+D2) grouped housed DHM, (Iso2+Veh2) socially isolated vehicle, (Iso2+D2) socially isolated DHM, n=4–5 per group. * = p≤ 0.05 vs. group housing control (G2+Veh2), n=5/group.

Corticosterone is the primary hormone of the pituitary adrenocortical axis in response to environmental challenges, and it has an essential function in stress (37). Corticosterone level was evaluated after social isolation to illustrate the effect of social isolation on the hypothalamic–pituitary–adrenal (HPA) axis. Our results revealed a significant increase in the serum corticosterone level from 285.8±26.65 ng/ml in G2+Veh2 to 466.0±60.51 ng/ml in Iso2+Veh2 group (F (3, 15) = 7.897), p = 0.002) (Fig. 5A). Administration of DHM at the dose of 2 mg /kg in the isolation group decreased the corticosterone level to 283.3±27.43 ng/ml closed to the basal level of G2+Veh2 ((Table 4S). This observation suggests that corticosterone responds to social isolation-induced stress challenges, and DHM attenuates the stress (anxiety) following social isolation and reduces corticosterone levels.

To determine the effect of social isolation on inflammation, we examined the protein expression level of NF-κB p65 utilizing Western blot. NF-κB transcription factor is an essential inflammatory pathway that plays a crucial role in regulating immune and inflammatory responses by inducing the expression of cytokines and chemokines genes (38). The active phosphorylated NF-κB p65 expression was significantly higher in the Iso2+Veh2 group compared to the control. DHM treatment group (Iso2+D2) shows a comparable phospho-NF-κBp65 protein level to the control (G2+Veh2) and treatment control (G2+ D2) (Fig 5B) ((*p* = 0.574). An increase in the expression of several numbers of proinflammatory cytokines and chemokines was observed using cytokines profile proteome assay in the isolated group (Iso2+ Veh2) compared to the control and DHM group-housed (G2+Veh2) (G2+ D2) (Fig 5C). The DHM treated isolated group (Iso2+D2) showed lower cytokines and chemokines expression levels compared to (Iso2+Veh2) (Table 2S). These data suggest that social isolation activates the NF-κB signaling pathway leading to an increase in the transcription of proinflammatory cytokines while DHM treatment counteract them.

## Discussion

The primary goal of this study was to determine the effects of anxiety induced by short-term social isolation in neuroinflammation and to identify potential pharmacological mechanisms underlying the therapeutic effects of DHM. In this study, we found that social isolation induced anxiety-like behaviors in mice, increased corticosterone level, decreased gephyrin expression, enhanced the activation of NF-kB p65 pathway, decrease hippocampal microglial cell number, and increase the reactive microglia. Importantly, these pathological changes and behavioral deficits were ameliorated by DHM treatment. Findings from the present work suggests that short-term social isolation leads to changes in the HPA axis, disrupts GABAergic neurotransmitters, and provokes neuroinflammation. DHM was found to restore these molecular and cellular changes.

Gephyrin is a key protein that anchors, clusters, and stabilizes GABAergic synapses (39). Mounting evidence has indicated the critical role of GABA transmission in the progression of anxiety disorder (40). In our previous study, we found that changes in gephyrin expression could reflect changes in GABA_A_R amount and GABAergic function (10, 33). Additionally, we found a reduction in GABA_A_R-mediated extra-synaptic, pre-, and post-synaptic tonic currents in the social isolation anxiety model, which was restored after DHM treatment (33). To elucidate the mechanism in which DHM modulates GABA_A_Rs in the socially isolated model, gephyrin protein expression was assessed utilizing Western blot. Gephyrin protein expression was reduced 40% in the isolation group and improved after DHM treatment (Fig. 1A). Taking into consideration that GABA_A_R expression is ubiquitous in the brain and the primary role of GABA neurotransmitter in anxiety pathogenesis, a reduction in gephyrin protein level might provide a partial explanation of the behavioral changes shown in (Fig 1B). It is essential to emphasize the interconnection between GABA and neuroinflammation. Neuroinflammation decreases GABA synthesis by reducing glutamate acid decarboxylase 67 (GAD67) enzyme, downregulating GABA_A_R protein expression, and inhibiting GABA current by decrease GABA neurons density (41). Therefore, studies of neuroinflammation will further clarify the underlining mechanism of DHM in improving anxiety-like behaviors.

As part of the innate immune system, microglia play a significant role in initiating and mediating neuroinflammation in the brain (42). Microglia are not only involved in brain infection and debris phagocytosis, but also play a crucial role in the physiological development of the brain by engaging in the shaping process of neuronal circuits and synapse plasticity (19, 20). Microglia activation occurred after various types of stress (21, 43, 44). We assessed microgliosis after short-term social isolation using immunohistochemistry staining of Iba1, one of the most common markers of microglia (45–47). These data indicate that social isolation-induced anxiety led to a decline in microglia cell number (Fig. 3C). Several evidence demonstrate a tight connection between the microglial loss mediated by overactivation and social isolation and other form of stress (48–50). Additionally, social isolation triggers microglia activation, manifested as changes in the cell morphology. High lacunarity value indicates the heterogeneity of the cells, meaning the single-cell image of microglia has different gap sizes. On the other hand, a low lacunarity value indicates homogeneity, suggesting the cell shape has less variance (36) . Under the influence of social isolation, the microglial cell showed a lower lacunarity value compared to the control. This implies the transformation of the microglial cell to a more homogenous state. This change generally suggests activation of the microglia from its surveillant state (51). Furthermore, the activated microglia cells in socially isolated mice showed a reduction in cell size, based on the measurement of the perimeter of single cell outlines. Reduction of cell perimeter is an illustration of fewer ramifications, shorter and thicker branches, and a larger soma size of the cell that manifested during activation (52). Microglia density is another morphometric parameter that was altered after social isolation and DHM treatment. Higher cell density demonstrates the transformation of the cells to a more compact form during activation (53, 54). The maximum span across the hull in each microglial cell was significantly smaller in the social isolation group than the control, reflecting the size of the overall cells and the length of the processes. Microglial in less ramified form is considered activated or intermediate active, which explains the reduction in the hull span size (51, 55). These results are consistent with the findings from several groups that demonstrated the activated microglia contribute to anxiety induced by social isolation (56, 57). In addition, several studies showed that microgliosis mediate synapses loss that is considered the earliest manifestation of Alzheimer’s disease (AD) pathology (58–60).

Accumulated evidence suggests a link between neuroinflammation and the pathology of anxiety and other psychological disorders (44, 61). Neuroinflammation is involved in several pathological changes in the nervous system and the neuroendocrine system, and it is initiated as a result of the modulation of the HPA axis (62). Chronic exposure to stress leads to disruption in HPA axis and an increase in the cortisol level in humans and corticosterone in rodents (63). Animal studies of the social isolation model have shown that the corticosterone level is higher after exposure to stress than the group-housed control (64). There was an increase in serum corticosterone level after 4 weeks of social isolation; and our results demonstrate that DHM treatment at doses of 2 mg/kg reduces corticosterone levels.

The transcription factor NF-κB plays a key function in regulating immune and inflammatory responses by inducing the expression of cytokines and chemokines genes (38). Growing evidence suggests that stress-induced behavioral deficits are mediated by NF-κB signaling activation, which increases the production of the proinflammatory cytokine and chemokines leading to microglia activation and ultimately neuroinflammation (65, 66). Our results indicate an increase in the Phospho-NF-kB p65 protein level compared to the nonactive NF-kB p65, which might explain the increase of proinflammatory cytokines expression, and the microglial activation observed in the social isolation group. Additionally, DHM treatment attenuates the activation of NF-κB pathway induced by social isolation, and this finding is consistent with a previous work that demonstrated a reduction in NF-κB protein expression after DHM treatment (67).

## Conclusion

There has been a growing body of evidence supporting the association of anxiety with cognitive decline leading researchers to conclude that anxiety symptoms could be predictive for the progression of Alzheimer’s disease (AD). This work provided insights into the mechanisms that DHM reverses the neuropathology resulted from anxiety as a component of preclinical AD, as well as the utility of DHM as a novel therapy for anxiety. DHM reduces neuroinflammation, restores GABAergic function by restoring gephyrin levels, and decreases serum cortisol levels, therefore, improves the social isolation induced anxiety and the early onset of AD. DHM could provide an early intervention to manage anxiety and decrease the chance of cognitive decline and AD development.

## Supplementary Material

Supplement 1

## Data Availability

All data generated or analysed during this study are included in this published article and in the supplementary information files.

## Abbreviations

DHM: Dihydromyricetin
NF-κB: Nuclear factor-κB
CNS: Central nervous system
PTSD: Posttraumatic stress disorder
GABA_A_Rs: GABA_A_ receptors
PAM: Positive allosteric modulator
HPA: hypothalamic-pituitary-adrenal
MAPK: Mitogen-activated protein kinase
BSA: Bovine serum albumin
PBS: Phosphate buffer saline
TBST: Tris-buffered saline with Tween 20
ELISA: Enzyme Linked Immmunosorbent Assay
ANOVA: Analysis of variance
EPM: Elevated plus-maze
OF: Open field
GAD67: Decarboxylase 67
AD: Alzheimer’s disease
