# Supplementary material for: Social Isolation Induces Neuroinflammation And Microglia Overactivation, While Dihydromyricetin Prevents And Improves Them
Source: Res Sq. 2021 Oct 1:rs.3.rs-923871. Preprint. [Version 1] doi: 10.21203/rs.3.rs-923871/v1 (PMC8491854; doi:10.21203/rs.3.rs-923871/v1)
Supplement: Supplement 1 [file 0f1595ff6a2820a424b54fba.docx]

**Table S1: One-way ANOVA and Holm-Sidak tests summary of the behavioral data**

**One-way ANOVA table**

|  |  | **SS** | **DF** | **MS** | **F (DFn, DFd)** | | **P value** |
| --- | --- | --- | --- | --- | --- | --- | --- |
| **Open arm** | Treatment (between columns) | 6.906 | 3 | 2.302 | | F (3, 39) = 3.721 | P=0.0191 |
|  | Residual (within columns) | 24.13 | 39 | 0.6187 | |  |  |
|  | Total | 31.03 | 42 |  | |  |  |
|  |  |  |  |  | |  |  |
| **Close arm** | Treatment (between columns) | 6.104 | 3 | 2.035 | | F (3, 29) = 3.001 | P=0.0466 |
|  | Residual (within columns) | 19.66 | 29 | 0.6780 | |  |  |
|  | Total | 25.76 | 32 |  | |  |  |

**Holm-Sidak test**

| **G2+Veh2** | **G2+D2** | **Iso2+Veh2** | **Iso2+D2** |
| --- | --- | --- | --- |

|  | **Mean ± SEM** | **N** | **Mean ± SEM** | **N** | **Mean ± SEM** | **N** | **Mean ± SEM** | **N** |
| --- | --- | --- | --- | --- | --- | --- | --- | --- |
| **Open arm** | 2.31± 0.27 | 11 | 2.3±0.26 | 11 | 1.26±0.17 | 10 | 2.07±0.22 | 11 |
| **Close arm** | 2.24±0.31 | 11 | 2.269±0.26 | 11 | 3.31±0.27 | 10 | 2.56±0.28 | 11 |
| **Running** | 2765±161.2 | 11 | 2873±141.2 | 11 | 2176±145.9 | 10 | 2398±146.9 | 11 |
| **Rearing** | 46.63±1.52 | 11 | 47.25±2.085 | 11 | 28.25±2.068 | 10 | 40.75±1.03 | 11 |
| **Centre** | 19.63±0.71 | 11 | 21.25±0.64 | 11 | 7.63±0.86 | 10 | 18.38±0.49 | 11 |
| **Corner** | 28.25±2.07 | 11 | 27.75±2.11 | 11 | 73.00±4.31 | 10 | 38.50±3.52 | 11 |

**Table 2S: Summary data of Western blot**

**One-way ANOVA table**

|  | **F** | **Sig.** | ***P* value** |
| --- | --- | --- | --- |

| **Gephyrin** | 29.02 | * | 0.0008 |
| --- | --- | --- | --- |
| **pNF-κB p65** | 4.401 | ns | 0.0222 |
| **NF-κB p65** | 0.5559 | ns | 0.6540 |

**Holm-Sidak test**

| **G2+Veh2** | **G2+D2** | **Iso2+Veh2** | **Iso2+D2** |
| --- | --- | --- | --- |

|  | | | |  | |  |  | | |  |  | | |  |  | |  | |
| --- | --- | --- | --- | --- | --- | --- | --- | --- | --- | --- | --- | --- | --- | --- | --- | --- | --- | --- |
|  | | **Mean ±SEM** | **N** | | **Mean ± SEM** | | | **N** | **Mean ± SEM** | | | **N** | **Mean ± SEM** | | | **N** | |  |
| **Gephyrin** | | 100± 3.522 | 3 | | NA | | | NA | 62.20±3.926 | | | 3 | 85.30±3.118 | | | 3 | |  |
| **pNF-κB p65** | | 0.5421±0.147 | 4 | | 0.5532±0.1098 | | | 4 | 0.9967±0.06064 | | | 5 | 0.4908±0.1382 | | | 5 | |  |
| **NF-κB p65** | | 1.13±0.09 | 4 | | 1.049±0.09 | | | 4 | 0.96±0.13 | | | 4 | 0.99±0.04 | | | 4 | |  |
|  |  |  |  |  |  |  |  |  |  |  |  |  |  |  |  |  |  |  |
|  |  |  |  |  |  |  |  |  |  |  |  |  |  |  |  |  |  |  |

**Table 3S: summary of microglia analysis data**

**One-way ANOVA table**

|  | **F** | **Sig.** | ***P* value** |
| --- | --- | --- | --- |

| **Lacunarity** | 3.828 | * | 0.0412 |
| --- | --- | --- | --- |
| **Perimeter** | 17.72 | * | <0.0001 |
| **Density** | 3.388 | * | 0.0487 |
| **Span across hull** | 14.36 | * | <0.0001 |
| **Microglia cell count** | 4.871 | * | 0.0145 |

**Holm-Sidak test**

| **G2+Veh2** | **Iso2+Veh2** | **Iso2+D2** |
| --- | --- | --- |

|  | | |  | |  |  | | | |  |  | | | | |  |  | |  | |
| --- | --- | --- | --- | --- | --- | --- | --- | --- | --- | --- | --- | --- | --- | --- | --- | --- | --- | --- | --- | --- |
|  | **Mean ±SEM** | **N** | | **Mean ± SEM** | | | **N** | | **Mean ± SEM** | | | **N** | |  |  |  |  |  |  |  |
| **Lacunarity** | 0.35± 0.2 | 7 | | 0.30±0.009 | | | 7 | | 0.34±0.01 | | | 7 | |  |  |  |  |  |  |  |
| **Perimeter** | 463.5±27.9 | 10 | | 278.7±16.76 | | | 11 | | 382.9±21.56 | | | 10 | |  |  |  |  |  |  |  |
| **Density** | 0.120±0.007 | 10 | | 0.144±0.006 | | | 11 | | 0.126±0.006 | | | 10 | |  |  |  |  |  |  |  |
| **Span across hull** | 172.5±12.04 | 10 | | 103.2±6.40 | | | | 11 | 140.0±8.84 | | | | 10 | |  | | |  | |  |
| **Microglia cell count** | 310.6±17.52 | 5 | | 237.5±13.74 | | | | 5 | 260.6±19.59 | | | | 5 | |  | | |  | |  |

**Table 4S: summary of corticosterone ELISA data**

**One-way ANOVA table**

|  | **F** | **Sig.** | ***P* value** |
| --- | --- | --- | --- |

| **Corticosterone** | 7.897 | * | 0.0022 |
| --- | --- | --- | --- |

**Holm-Sidak test**

| **G2+Veh2** | **G2+D2** | **Iso2+Veh2** | **Iso2+D2** |
| --- | --- | --- | --- |

|  | | |  | |  |  | | |  |  | | |  |  | |  | |
| --- | --- | --- | --- | --- | --- | --- | --- | --- | --- | --- | --- | --- | --- | --- | --- | --- | --- |
|  | **Mean± SEM** | **N** | | **Mean ± SEM** | | | **N** | **Mean ± SEM** | | | **N** | **Mean ±SEM** | | | **N** | |  |
| **Corticosterone** | 285.8±26.65 | 5 | | 240.1±19.98 | | | 5 | 466.0±60.51 | | | 4 | 283.3±27.43 | | | 5 | |  |
